# The role of the osmosensitive transcription factor NFAT5 in corneal edema resorption after injury

**DOI:** 10.1038/s12276-023-00954-w

**Published:** 2023-03-03

**Authors:** Karina Hadrian, Gwen Musial, Alfrun Schönberg, Tihomir Georgiev, Christoph Küper, Felix Bock, Jonathan Jantsch, Claus Cursiefen, Sabine A. Eming, Deniz Hos

**Affiliations:** 1 Department of Ophthalmology, Faculty of Medicine and University Hospital Cologne, University of Cologne, 50937 Cologne, Germany; 2 Center for Molecular Medicine Cologne (CMMC), University of Cologne, 50937 Cologne, Germany; 3 Institute for Molecular Medicine, MSH Medical School Hamburg, 20457 Hamburg, Germany; 4 Institute for Medical Microbiology, Immunology, and Hygiene, University Hospital Cologne and Faculty of Medicine, University of Cologne, 50937 Cologne, Germany; 5 Department of Dermatology, University of Cologne, 50937 Cologne, Germany; 6 Cologne Excellence Cluster on Cellular Stress Responses in Aging-Associated Diseases (CECAD), University of Cologne, 50937 Cologne, Germany; 7 Developmental Biology Unit, Institute of Zoology, University of Cologne, 50937 Cologne, Germany

**Keywords:** Immunology, Cell biology

## Abstract

The osmosensitive transcription factor nuclear factor of activated T cells 5 (NFAT5; or tonicity-responsive enhancer binding protein; TonEBP) plays a key role in macrophage-driven regulation of cutaneous salt and water balance. In the immune-privileged and transparent cornea, disturbances in fluid balance and pathological edema result in corneal transparency loss, which is one of the main causes of blindness worldwide. The role of NFAT5 in the cornea has not yet been investigated. We analyzed the expression and function of NFAT5 in naive corneas and in an established mouse model of perforating corneal injury (PCI), which causes acute corneal edema and transparency loss. In uninjured corneas, NFAT5 was mainly expressed in corneal fibroblasts. In contrast, after PCI, NFAT5 expression was highly upregulated in recruited corneal macrophages. NFAT5 deficiency did not alter corneal thickness in steady state; however, loss of NFAT5 led to accelerated resorption of corneal edema after PCI. Mechanistically, we found that myeloid cell-derived NFAT5 is crucial for controlling corneal edema, as edema resorption after PCI was significantly enhanced in mice with conditional loss of NFAT5 in the myeloid cell lineage, presumably due to increased pinocytosis of corneal macrophages. Collectively, we uncovered a suppressive role for NFAT5 in corneal edema resorption, thereby identifying a novel therapeutic target to combat edema-induced corneal blindness.

## Vision: Potential therapeutic target for corneal blindness

A protein called nuclear factor of activated T cells 5 (NFAT5) plays a significant role in maintaining fluid balance in the cornea, the anterior part of the eye. Disturbances in corneal fluid balance and pathological swelling known as edema potentially lead to loss of corneal transparency and blindness. Deniz Hos and colleagues at the University of Cologne, Germany, studied the functional role of NFAT5 in a mouse model of corneal injury. They found that corneal injury induced significantly increased NFAT5 activity. Furthermore, NFAT5 in macrophages was crucial for controlling corneal fluid balance and the extent of and recovery from corneal edema. These results suggest that drugs able to modify NFAT5 activity could offer a novel way to combat edema-induced corneal blindness.

## Introduction

The cornea is the transparent avascular outer barrier and major refractive element of the eye^1^. Loss of corneal transparency leads to corneal blindness and is the second most common cause of blindness worldwide. The only possible treatment thus far is corneal transplantation, resulting in more than a million people suffering from corneal blindness due to a shortage of donor corneas. In industrialized countries, dysfunction of corneal endothelial cells, which leads to corneal swelling (edema) and consecutive loss of transparency, is one of the main indications for corneal transplantation^2,3^. Thus, a nonsurgical approach to reduce corneal edema would be of great therapeutic value to treat corneal blindness worldwide.

The cornea contains no blood or lymphatic vessels in its healthy state, which is called the (lymph)angiogenic privilege of the cornea^1^. However, severe injury or inflammation can overcome this (lymph)angiogenic privilege, leading to the secondary ingrowth of blood and lymphatic vessels into the cornea^4–7^. The ingrowth of lymphatic vessels is critically involved in the development of allograft rejection after corneal transplantation, as lymphatic vessels ease the trafficking of antigen-presenting cells from the corneal allograft to the secondary lymphatic organs. However, we have recently also demonstrated a beneficial role for lymphatic vessels in the cornea^8,9^. Specifically, we showed that injury-associated corneal edema leads to transient corneal lymphangiogenesis, which is mediated by macrophages and regulates the resolution of corneal edema and the restoration of corneal transparency^9,10^.

Recently, it was demonstrated that lymphangiogenesis plays an important role in the salt/water balance in the skin^11,12^. It has been shown that a high-salt diet can lead to cutaneous hypertonic Na^+^ accumulation^13–15^, which is paralleled by enhanced lymph flow in skin and muscle. Of note, high-salt diets trigger the secretion of vascular endothelial growth factor C (VEGF-C) from skin macrophages, increasing the density of the lymphatic vasculature and thereby enhancing electrolyte clearance^11,12,16^. The underlying molecular pathway involves the activation of nuclear factor of activated T cells 5 (NFAT5; or tonicity-responsive enhancer binding protein; TonEBP), an osmosensitive transcription factor that directly regulates VEGF-C secretion by skin macrophages^11^. In addition to being activated by osmotic changes, NFAT5 in macrophages has been shown to be induced by proinflammatory stimuli (reviewed in:^17^).

Although the involvement of NFAT5 in corneal diseases associated with disturbances in corneal salt/water balance and edema is conceivable, this has not been investigated yet. In this work, we therefore studied whether NFAT5 contributes to the regulation of corneal fluid balance at rest and in the resolution after injury.

## Materials and methods

### Mouse model of perforating corneal injury (PCI)

PCI was performed as previously described^9^. In brief, mice received atropine sulfate eye drops (Atropin POS 1%, Ursapharm GmbH, Saarbruecken, Germany) prior to surgery to avoid iris incarceration. Then, a perforating corneal incision 1 mm in length was generated using a 30-gauge needle and surgical microscissors. After PCI, mice received ofloxacin eye drops (Ofloxacin, Ratiopharm GmbH, Ulm, Germany) two times daily for 3 days.

### Immunohistochemistry of corneal whole mounts

Corneas were removed and immediately fixed with acetone, followed by blocking in 2% BSA. Corneas were stained with Alexa Fluor-647 anti-vimentin (Abcam, Cambridge, UK), anti-F4/80 (Invitrogen, Eugene, USA), anti-NFAT5 (Thermo Fisher, USA) or anti-LYVE1 (Abcam, UK). Secondary antibodies for staining included Alexa Fluor 555 goat anti-rat and Alexa Fluor 488 goat anti-rabbit (Invitrogen, USA).

### Evaluation of NFAT5 colocalization with vimentin and F4/80

Corneal whole mounts were analyzed at 400-fold magnification using an LSM 880 Confocal Microscope (Zeiss, Jena, Germany). Stack images of the corneal stroma were analyzed using MATLAB software (The MathWorks, Inc., Natick, MA). The single-channel images were loaded into the software, and the Pearson correlation coefficient was calculated for each image set.

### Cultivation of bone marrow-derived macrophages

Bone marrow-derived macrophages (BMDMs) were isolated according to standard protocols. Cells were cultured for 7 days in DMEM (Life Technologies, Eugene, USA) containing 10% FBS (Life Technologies, Eugene, USA) in the presence of 20 ng/ml M-CSF (Miltenyi Biotec, Bergisch Gladbach, Germany). Afterward, macrophages were costimulated with 10 ng/ml LPS and 100 ng/ml IFN-γ for 24 h.

### RNA isolation and real-time PCR analysis

On day 7 after PCI, central corneas sparing the limbal area were excised, and RNA was isolated using RNeasy Micro Kit (Qiagen, Hilden, Germany). RNA isolation was performed according to manufacturer’s instructions, including DNAse treatment (for 10 min). The RNA concentration and purity were assessed using Nanodrop 2000c Spectrometer (Thermo Scientific, Waltham, MA, USA). Complementary DNA (cDNA) synthesis was performed in random hexamer primed reverse transcriptase reactions (SuperScript III; Invitrogen, Carlsbad, CA) according to manufacturer’s instructions. Real-time PCR analysis was performed with 20 ng of cDNA using the SsoFast™ EvaGreen® Supermix (Bio-Rad Laboratories, Inc. Hercules, CA, USA) with a final primer concentration of 0.33 pmol/µl. Primers (Eurofins Genomics, Ebersberg, Germany) were designed using Primer3 software (https://primer3.ut.ee) and Basic Local Alignment Search Tool (National Center for Biotechnology Information, Bethesda, MD). Primer sequences for real-time PCR and annealing temperatures are provided in Table 1. Real-time PCRs were performed using CFX96® Real-Time System (Bio-Rad Laboratories, Inc. Hercules, CA, USA). A subsequent melt curve analysis was performed to check the amplification specificity. All PCR products were additionally analyzed by gel electrophoresis on a 2% agarose gel. The results were analyzed by the comparative threshold cycle method with hypoxanthine-guanine phosphoribosyltransferase (HPRT) as the endogenous reference gene for all reactions. All assays were performed in triplicate, and a nontemplate control was included in all experiments to exclude DNA contamination. The temperature profile of the PCR is given in Table 2.

**Table 1:** Primer sequences used in qPCR experiments.

Gene	Sequences	Annealing Temperature
*Hprt*	For: 5'-GTTGGATACAGGCCAGACTTTGTTG-3' Rev: 5'-GATTCAACTTGCGCTCATCTTAGGC-3'	60–63 °C
*Nfat5*	For: 5'-AACATTGGACAGCCAAAAGG-3' Rev: 5'-GCAACA CCA CTG GTT CAT TA-3'	60 °C
*Vegfc*	For: 5'-AGAACGTGTCCAAGAAATCAGC-3' Rev: 5'-ATGTGGCCTTTTCCAATACG-3'	60 °C
*Aqp1*	For: 5'-CAGCAAGTCCCCCACCTTAG-3' Rev: 5'-TAAGGTGCCTACCCCAAGGA-3'	60 °C
*Aqp5*	For: 5'-CAAAGCCTTCCCCCAGGTAG-3' Rev: 5'-AAGATGGCACTCGACGAACC-3'	60 °C

**Table 2:** Temperature profile for qPCR.

Step	Temperature	Time	
Initial denaturation	95 °C	3:00 min	
Denaturation	95 °C	00:10 min	Repeat 45x
Annealing	See Table 1 for specific temperature	00:30 min	
Melt Curve	65 °C to 96 °C	Increment 0.5 °C for 00:05 min	

### Mouse models

The tamoxifen-inducible, ubiquitous NFAT5 knockout mouse model used in this study was originally generated by Küper et al.^18^. To induce Cre-mediated recombination, tamoxifen was thoroughly dissolved at 37 °C in ethanol (200 mg/mL; Sigma Aldrich, Schnelldorf, Germany), diluted with corn oil (vehicle) to a final concentration of 20 mg/mL, and administered intraperitoneally (200 μg/g body weight) two times with an interval of 48 h between injections. UbcCre^+^/NFAT5^fl/fl^ and UbcCre^-^/NFAT5^fl/fl^ mice (both on a C57BL/6 background) were injected with tamoxifen. To exclude the effects of tamoxifen that are not related to Cre-mediated recombination, additional experiments comparing tamoxifen-injected UbCCre^-^/NFAT5^fl/fl^ mice with vehicle-injected UbCCre^-^/NFAT5^fl/fl^ mice were performed. Mice were analyzed 4 weeks after the final day of tamoxifen administration to ensure clearance of the targeted protein. If mice underwent surgery, they were injected 1 day presurgery with 100 µg/g body weight tamoxifen.

The myeloid cell-specific NFAT5 knockout mouse model was generated by crossing LysMCre mice^19^ with NFAT5^fl/fl^ mice (both on a C57BL/6 background). Genotyping protocols for both mouse lines are given in the Supplementary Material.

### Assessment of mean corneal thickness (MCT)

MCT as a measure of corneal edema was analyzed using in vivo optical coherence tomography (OCT) volume scans of the anterior segment using Telesto OCT Systems (Thorlabs, Lübeck, Germany). B-Scans were acquired with a scan size of 512 × 512 pixels covering an area of 2.5 mm × 2.5 mm with an imaging depth of 1.87 mm. The scaling of 3.65 µm/pixel and 4.88 µm/pixel in the axial and lateral directions, respectively, was calculated using the refractive index of the cornea (1.376). The mean corneal thickness was assessed using MATLAB software (The MathWorks, Inc., Natick, MA). The volume of OCT scans was loaded into MATLAB and divided into 10 frame stacks. For each stack, a maximum intensity projection (MIP) image was created. First, the lens was detected by a multithresh function and removed from the image. The MIP image was contrast enhanced followed by a median filter. Then, edge detection was performed with a Sobel filter, and the top and bottom edges of the cornea were segmented. The gradient of the edge segmentations was calculated, and points that had high derivatives were removed. The missing points of the cornea edge segmentation were then linearly extrapolated, followed by optional manual modification. The corneal thickness was determined by the distance between the top and bottom edge segmentations, and the software rejected any thickness values that were greater than or less than two standard deviations of the mean thickness for that MIP image. The average thickness across each MIP image was found, and the thickness for the whole cornea volume was taken to be the average of the thicknesses from the MIP images.

### Pinocytosis analysis in bone marrow-derived macrophages

Pinocytosis was assessed using 30 µg/ml pHrodo^TM^ Green Dextran (Live Technologies, Carlsbad, CA, USA) according to the manufacturer’s instructions. In brief, activated BMDMs were incubated with 30 µg/ml pHrodo^TM^ Green Dextran in Live Cell Imaging Solution (Live Technologies, Carlsbad, CA, USA). Fluorescence and phase contrast images were taken every 20 min for 4 h using IncuCyte Zoom (Essen Bioscience, Hertfordshire, UK). For the evaluation of saturated cells, images of the fluorescent cells were loaded into MATLAB software (The MathWorks, Inc., Natick, MA) for saturated cell counting. First, the background signal in the image was subtracted using a rolling ball algorithm. Then, the image was converted to binary with a set threshold based on the saturation intensity value. Objects <10 pixels were removed, and the remaining cells were automatically counted. A phase contrast image was used to count the cells, and the relative number of saturated cells was calculated.

### Statistical analyses

Significance was analyzed using Student’s t test for parametric data or the Mann‒Whitney U test for nonparametric data. If more than two groups were compared, one-way ANOVA with multiple comparisons was performed. All data are presented as grouped values with the mean and standard deviation (SD). A value of *p* < 0.05 was considered statistically significant.

### Study approval

All animal experiments were approved by the local animal care committee (LANUV, Approval No. 84-02.04.2015.A487 and 81-02.04.2021.A110) and were performed in accordance with the Association for Research in Vision and Ophthalmology Statement for the Use of Animals in Ophthalmic and Vision Research.

## Results

### NFAT5 is differentially expressed in naive corneas after perforating corneal injury

**Fig. 1:**
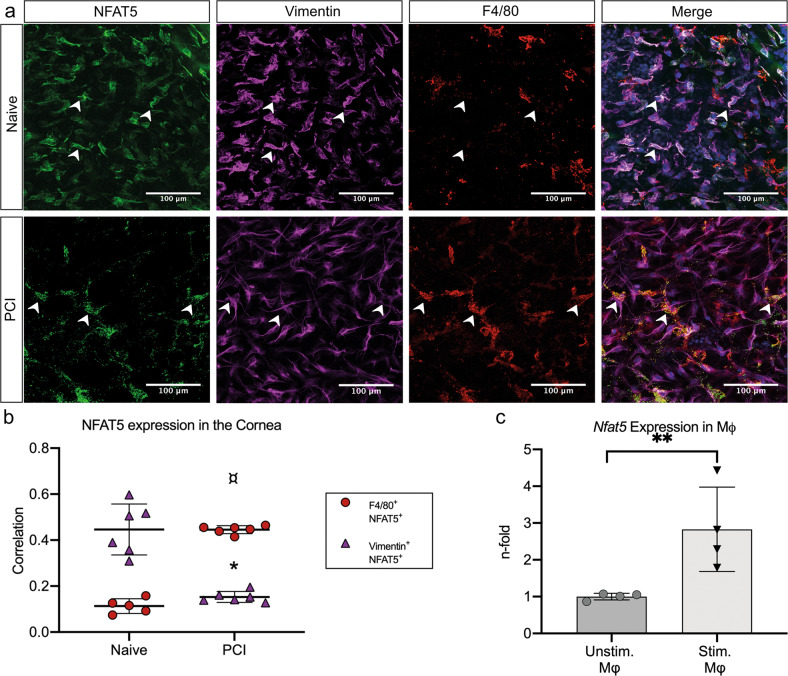
Differential expression of NFAT5 in naive corneas and corneas after perforating corneal injury (PCI). **a** Expression of NFAT5 (green) in naive (top) and injured (bottom) corneal whole mounts. Vimentin (purple) was used as a marker for corneal fibroblasts, and F4/80 (red) was used as a marker for corneal macrophages. Magnification: 40x, arrowheads indicate coexpression of NFAT5 with vimentin or F4/80. **b** Quantification of the coexpression of NFAT5 with vimentin or F4/80 in naive and injured corneas (*indicates the statistical significance between vimentin^+^/NFAT5^+^ cells in naive vs. injured corneas; ¤ indicates the statistical significance between F4/80 ^+^/NFAT5^+^ cells in naive vs. injured corneas (*n* = 6)). **c** mRNA expression of *Nfat5* in bone marrow-derived macrophages 24 h after stimulation with LPS/interferon-γ; **p < 0.01, *n* = 5.

We first analyzed NFAT5 expression in uninjured and injured corneas using confocal microscopy. We chose an established mouse model of perforating corneal injury (PCI), as this type of injury is associated with severe corneal edema^9^. In naive corneas (Fig. 1a), NFAT5 was mainly expressed in vimentin^+^ fibroblasts as well as in F4/80^+^ macrophages. Correlation analysis (Fig. 1b) of NFAT5 colocalization with either vimentin or F4/80 resulted in a high colocalization of NFAT5 and vimentin (correlation: 0.45 ± 0.11) in naive corneas, whereas the colocalization of NFAT5 and F4/80 was low (correlation: 0.11 ± 0.03). After PCI, however, colocalization of NFAT and vimentin significantly decreased (correlation: 0.15 ± 0.02; *p* < 0.0001), whereas colocalization of NFAT5 and F4/80 significantly increased (correlation: 0.45 ± 0.02; *p* < 0.0001). To better define the macrophage population that preferentially expresses NFAT5, we performed mRNA expression analyses of stimulated bone marrow-derived macrophages (BMDMs) in vitro. Here, we showed that NFAT5 expression was increased in lipopolysaccharide (LPS)- and interferon-γ (IFN-γ)-activated inflammatory macrophages (x2.83 ± 1.15; *p* < 0.01; Fig. 1c).

### F4/80^+^ cells in naive corneas are increased in ubiquitous NFAT5 knockout mice

**Fig. 2:**
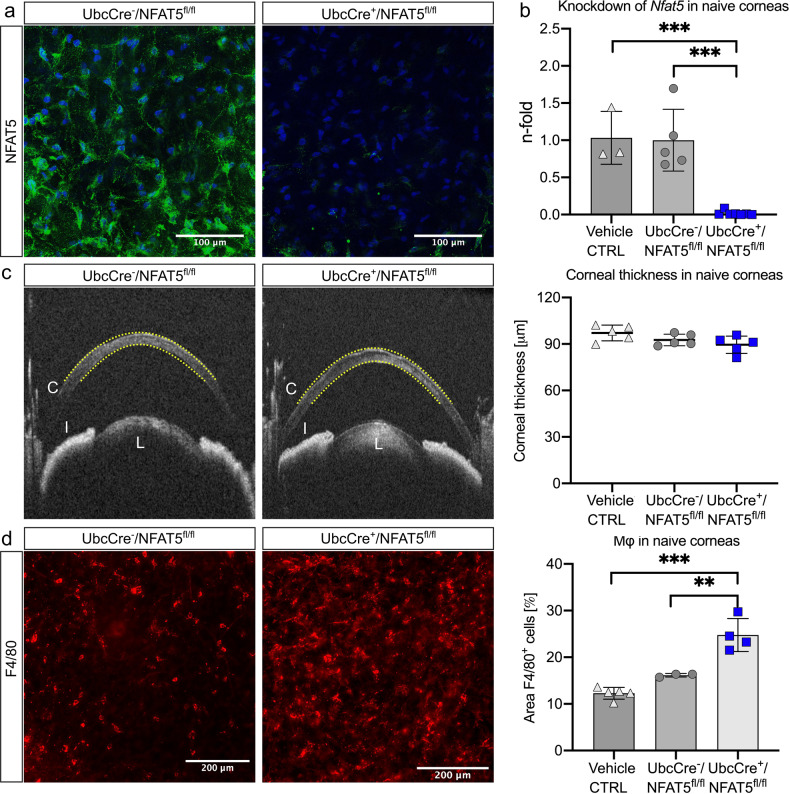
Ubiquitous knockout of NFAT5 leads to an increase in F4/80^+^ cells in naive corneas. **a** Tamoxifen-induced knockdown of NFAT5 in corneal whole mounts (*n* = 4); NFAT5 (green) and nuclear stain (blue); magnification: 40x. **b** Knockdown of *Nfat5* in the naive cornea assessed by qPCR (*n* = 5-6). **c** Corneal thickness after tamoxifen-induced knockdown of NFAT5 assessed by in vivo optical coherence tomography. Yellow dotted lines indicate the area of corneal thickness measurement. C cornea, I iris, L lens. **d** F4/80^+^ corneal macrophages (red) after tamoxifen-induced knockdown of NFAT5; magnification: 20x; the corneal area covered by F4/80^+^ cells in relation to the whole cornea was evaluated. **p < 0.01 (*n* = 3–4) Corn oil only without tamoxifen (vehicle control) was used as an additional control in all analyses.

To analyze the role of NFAT5 in corneal fluid homeostasis, we used a mouse model in which NFAT5 can be ubiquitously deleted by systemic application of tamoxifen (UbcCre/NFAT5^fl/fl^;^18^). Successful NFAT5 deletion was verified at protein and transcriptional levels using immunofluorescence (Fig. 2a) and qPCR (Fig. 2b) 4 weeks after tamoxifen application. Injection of vehicle only without tamoxifen had no effect on NFAT5 mRNA expression (Fig. 2b). To investigate whether loss of NFAT5 alters corneal fluid homeostasis in the steady state, the thickness of the cornea as a measure of corneal edema was evaluated using in vivo optical coherence tomography (OCT) volume scans of the anterior segment (Fig. 2c). The mean corneal thickness (MCT) was not altered among vehicle-injected UbcCre^-^/NFAT5^fl/fl^ controls (97.08 ± 4.54 µm), tamoxifen-injected UbcCre^-^/NFAT5^fl/fl^ controls (92.59 ± 3.76 µm) and tamoxifen-injected UbcCre^+^/NFAT5^fl/fl^ corneas (89.52 ± 5.59 µm). However, evaluation of F4/80^+^ cells in corneal whole mounts showed increased numbers of macrophages in corneas of tamoxifen-injected UbcCre^+^/NFAT5^fl/fl^ mice (corneal area covered by F4/80^+^ cells: 24.76 ± 3.54%) compared to tamoxifen-injected UbcCre^-^/NFAT5^fl/fl^ mice (16.14 ± 0.36%; *p* < 0.01; Fig. 2d). Vehicle-injected UbcCre^-^/NFAT5^fl/fl^ controls showed no significant alterations compared to tamoxifen-injected UbcCre^-^/NFAT5^fl/fl^ controls.

### Injury-induced corneal edema resorption is enhanced in ubiquitous and myeloid cell-specific NFAT5 knockout mice

**Fig. 3:**
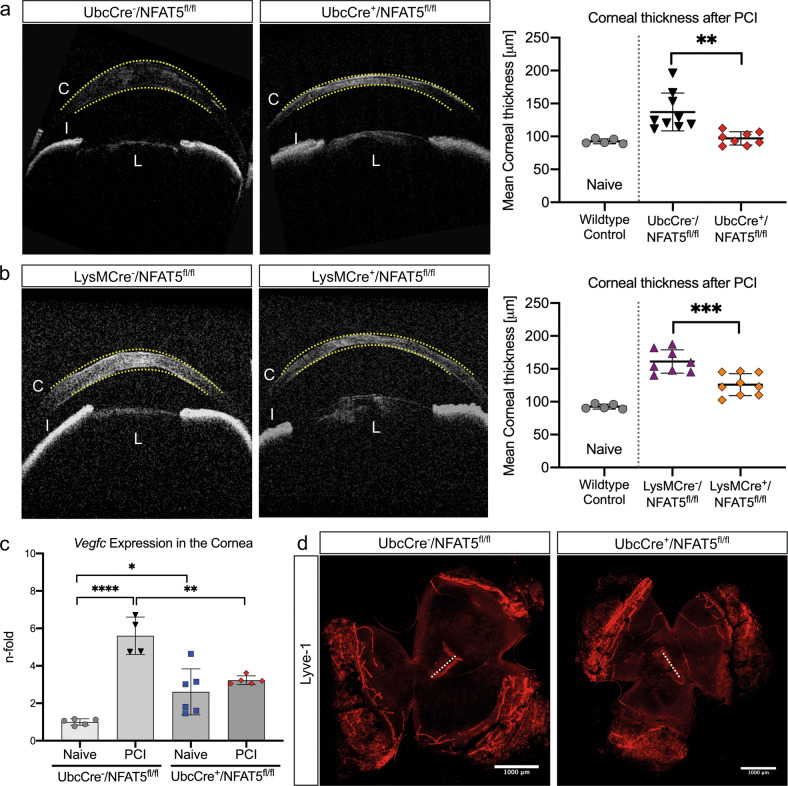
Ubiquitous and myeloid cell-specific NFAT5 knockout ameliorates injury-induced corneal edema resorption independent of lymphangiogenesis. Corneal thickness in (**a**) UbcCre^-^/NFAT5^fl/fl^ mice and UbcCre^+^/NFAT5^fl/fl^ mice (*n* = 8–9) and (**b**) LysMCre^-^/NFAT5^fl/fl^ mice (*n* = 8–9) and LysMCre^+^/NFAT5^fl/fl^ mice assessed by in vivo optical coherence tomography. Yellow dotted lines indicate the area of corneal thickness measurement. C cornea, I iris, L lens. **c** Whole corneal *Vegfc* mRNA expression in UbcCre^-^/NFAT5^fl/fl^ and UbcCre^+^/NFAT5^fl/fl^ mice after PCI (*n* = 5–6). **d** Lymphatic vessel staining in UbcCre^-^/NFAT5^fl/fl^ mice versus UbcCre^+^/NFAT5^fl/fl^ mice after PCI (LYVE-1; red); 10x magnification. The dotted line indicates the area of the PCI (*n* = 6). *p < 0.05; **p < 0.01; ***p < 0.001; ****p < 0.0001.

To investigate whether NFAT5 is functionally relevant in the regulation of corneal edema resolution after injury, we performed PCI in UbcCre/NFAT5^fl/fl^ mice and in LysMCre/NFAT5^fl/fl^ mice, in which NFAT5 is specifically deleted in myeloid cells. In injured UbcCre^-^/NFAT5^fl/fl^ mice, MCT was significantly increased 1 week after injury (137.1 ± 28.8 µm) compared to uninjured controls (89.52 ± 5.59 µm), as expected. Strikingly, in UbcCre^+^/NFAT5^fl/fl^ corneas, MCT after PCI was significantly lower (97.10 ± 10.0 µm; *p* < 0.01) than in injured UbcCre^-^/NFAT5^fl/fl^ corneas (Fig. 3a). A similar alteration pattern was found in mice with conditional loss of NFAT5 in the myeloid cell lineage (LysMCre/NFAT5^fl/fl^ mice): in injured LysMCre^-^/NFAT5^fl/fl^ mice, MCT was significantly increased 1 week after injury (161.3 ± 17.8 µm) compared to uninjured controls (92.59 ± 3.76 µm). Importantly, in LysMCre^+^/NFAT5^fl/fl^ corneas, MCT after PCI was significantly reduced (126.0 ± 16.6 µm; *p* < 0.001) compared to injured LysMCre^-^/NFAT5^fl/fl^ corneas (Fig. 3b). Thus, our results demonstrate that NFAT5 critically regulates corneal edema resorption after PCI.

It has been shown that NFAT5 regulates the expression of *Vegfc* in macrophages and lymphangiogenesis in the skin^11^. To test whether NFAT5 also regulates the expression of *Vegfc* in the cornea, corneas were analyzed by qPCR (Fig. 3c). Analysis of corneal mRNA of UbcCre^-^/NFAT5^fl/fl^ mice (Fig. 3c) revealed significantly increased expression of *Vegfc* in injured (x5.61 ± 0.55; *p* < 0.0001) versus uninjured (x1.00 ± 0.15) corneas, which is in line with previous results demonstrating an increase in *Vegfc* expression after PCI^9^. In uninjured UbcCre^+^/NFAT5^fl/fl^ mice, the expression of *Vegfc* was already significantly higher (x2.61 ± 0.49; *p* = 0.014) in uninjured UbcCre^-^/NFAT5^fl/fl^ controls (arguably due to increased macrophage numbers). However, the increase in *Vegfc* expression after PCI was less pronounced in the corneas of UbcCre^+^/NFAT5^fl/fl^ mice (x3.32 ± 0.52) than in those of UbcCre^-^/NFAT5^fl/fl^ mice. In fact, *Vegfc* expression in injured UbcCre^+^/NFAT5^fl/fl^ corneas remained statistically unchanged compared to uninjured UbcCre^+^/NFAT5^fl/fl^ corneas (*p* > 0.05). We next analyzed corneal lymphangiogenesis after PCI in UbcCre/NFAT5 mice. Interestingly, the ingrowth of lymphatic vessels, the diameter of vessels and the number of vessel sprouts after PCI were not altered in UbcCre^+^/NFAT5^fl/fl^ mice compared to UbcCre^-^/NFAT5^fl/fl^ mice (Fig. 3d).

Aquaporins (AQPs) belong to the family of transmembrane water channels and are involved in fluid homeostasis. In the cornea, two types of AQPs are expressed, namely, AQP1 and AQP5 (reviewed in:^20^). NFAT5 promotes the expression of AQP1 and AQP5 in E17.5 mouse embryos^21^. To investigate whether the observed differences in MCT in UbcCre^+^/NFAT5^fl/fl^ and LysMCre^+^/NFAT5^fl/fl^ mice might be a result of alterations in *Aqp1* and *Aqp5* mRNA expression, we investigated the mRNA expression levels of *Aqp1* and *Aqp5* in injured corneas of UbcCre^+^/NFAT5^fl/fl^ mice and UbcCre^-^/NFAT5^fl/fl^ mice. However, there was no difference in the expression of both *Aqp1* and *Aqp5* after PCI in UbcCre^+^/NFAT5^fl/fl^ mice compared to UbcCre^-^/NFAT5^fl/fl^ mice (Supplementary Fig. 1).

### The pinocytosis capacity of bone marrow-derived macrophages is suppressed by NFAT5

**Fig. 4:**
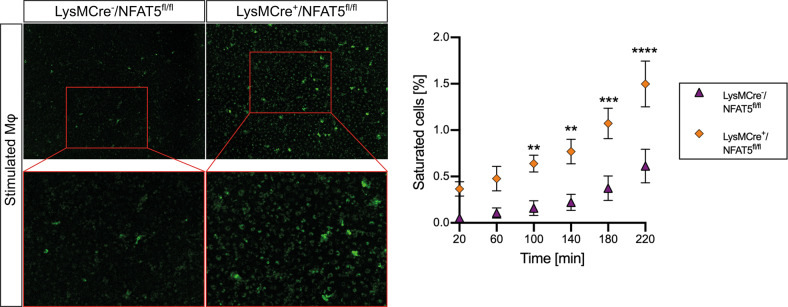
NFAT5 suppresses the pinocytosis capacity in bone-marrow derived macrophages. Representative images of dextran intake via pinocytosis in LPS/IFN-γ-costimulated bone marrow-derived macrophages (BMDMs) from LysMCre^-^/NFAT5^fl/fl^ and LysMCre^+^/NFAT5^fl/fl^ mice. Images were obtained every 40 min with 9 images per well, and the percentage of saturated cells in each image was calculated (*n* = 3). *p < 0.05; **p < 0.01; ***p < 0.001; ****p < 0.0001.

Our results indicate that NFAT5 deficiency promotes the resolution of corneal edema independent of corneal lymphangiogenesis, as lymphangiogenesis in UbcCre^+^/NFAT5^fl/fl^ mice was unaltered and independent of AQP expression. It is well established that leukocytes are capable of ingesting fluid, a special form of endocytosis called pinocytosis^22,23^. Because NFAT5 was mainly expressed by macrophages after PCI, we next investigated whether NFAT5 is involved in the regulation of pinocytosis in BMDMs obtained from LysMCre^-^/NFAT5^fl/fl^ and LysMCre^+^/NFAT5^fl/fl^ mice. After incubation with pHrodo^TM^ Green Dextran, the percentage of cells saturated with fluorescent dextran in LPS/IFN-γ-stimulated macrophages was significantly higher in LysMCre^+^/NFAT5^fl/fl^ macrophages than in LysMCre^-^/NFAT5^fl/fl^ macrophages (1.45 ± 0.28% vs. 0.61 ± 0.18%; *p* < 0.0001; Fig. 4). These results suggest that NFAT5 restricts the pinocytotic capacity of macrophages.

## Discussion

In this study, we present NFAT5 as a new factor regulating the resolution of corneal edema after PCI.

Our work has also demonstrated that an inflammatory stimulus introduced by PCI leads to an NFAT5 expression switch from corneal fibroblasts to macrophages. However, the molecular factors regulating this expression switch and its functional consequences are still unknown.

Next, we demonstrated that the loss of NFAT5 in naive corneas did not result in any alterations in MCT in naive UbcCre^+^/NFAT5^fl/fl^ corneas compared to UbcCre^-^/NFAT5^fl/fl^ corneas. However, corneas of UbcCre^-^/NFAT5^fl/fl^ and LysMCre^-^/NFAT5^fl/fl^ mice demonstrated severe edema after PCI in comparison to UbcCre^+^/NFAT5^fl/fl^ and LysMCre^+^/NFAT5^fl/fl^ mouse corneas, showing a significantly lower MCT, which was almost close to the thickness of uninjured corneas.

Our data allow us to propose a mechanism by which NFAT5 blockade is protective. First, we observed an increase in the number of macrophages in naive UbcCre^+^/NFAT5^fl/fl^ corneas. This is in contrast to renal tissues, where monocyte recruitment into the medulla is dependent on NFAT5^24^. However, these results were obtained in an injury-independent model, which could account for the observed differences from our model. Our data suggest that increased macrophage numbers in corneal tissue might contribute to the protective effect.

As shown previously, NFAT5 is a transcription factor for one of the main pro-lymphangiogenic growth factors, VEGF-C^11^, which ultimately regulates lymphatic vessel growth in skin via macrophages^12^. We have previously shown in PCI that corneal lymphatic vessels are induced by macrophages and that corneal lymphatics play a beneficial role in the resorption of corneal edema^9,10^.

In naive mice, however, loss of NFAT5 did not result in any obvious changes in the architecture of the corneal lymphatic vasculature, although we noted a small increase in corneal *Vegfc* expression in naive mice, which might be attributable to increased corneal macrophage numbers. In line with this reasoning, we reported earlier that there was a positive correlation between corneal macrophage numbers and corneal *Vegfc* expression^25,26^. Of note, in line with its role as a positive regulator of *Vegfc* expression^11,12^, NFAT5 contributed to increased *Vegfc* expression in injured corneal tissues, which did not, however, translate into obvious alterations in the lymphatic corneal network at the time points analyzed. Based on these findings, we conclude that NFAT5 deficiency-driven accelerated resorption of corneal edema is not linked to changes in lymphatic vessel architecture.

Pinocytotic fluid removal might play a role in edema resolution. We were able to show that the pinocytotic capacity of BMDMs generated from LysMCre^+^/NFAT5^fl/fl^ mice was significantly higher in BMDMs generated from LysMCre^-^/NFAT5^fl/fl^ mice. In line with this, a recent study has shown that deletion of NFAT5 promotes lipid uptake in vascular smooth muscle cells^27^, which also supports the observation that NFAT5 is a negative regulator of endocytosis. Moreover, since (a) pinocytotic activity is lower in inflammatory-stimulated macrophages and (b) NFAT5 contributes to proinflammatory macrophage activation^17,28–30^, our findings of enhanced pinocytosis might be due to reduced macrophage activation and a subsequently increased pinocytotic capacity that is preserved in less activated macrophages. Finally, NFAT5 fosters autophagy^31^. Cancer cells are able to overcome autophagy blockade by enhancing their (macro)pinocytotic capacity in a ‘Nuclear Factor Erythroid 2-related Factor 2’ (NRF2)-dependent manner. Therefore, it is tempting to speculate that NFAT5 deficiency might result in a compensatory Nrf2-dependent upregulation of pinocytotic activity in macrophages as well. Mechanistically, enhanced pinocytosis of extracellular fluids might facilitate the transport of fluids out of the corneal tissue. This proposed mechanism would be comparable to findings in brain ischemia models where there is increased basolateral pinocytosis by endothelial cells after brain ischemia in rats potentially facilitating removal of fluids from injured brain tissue^32^.

**Fig. 5:**
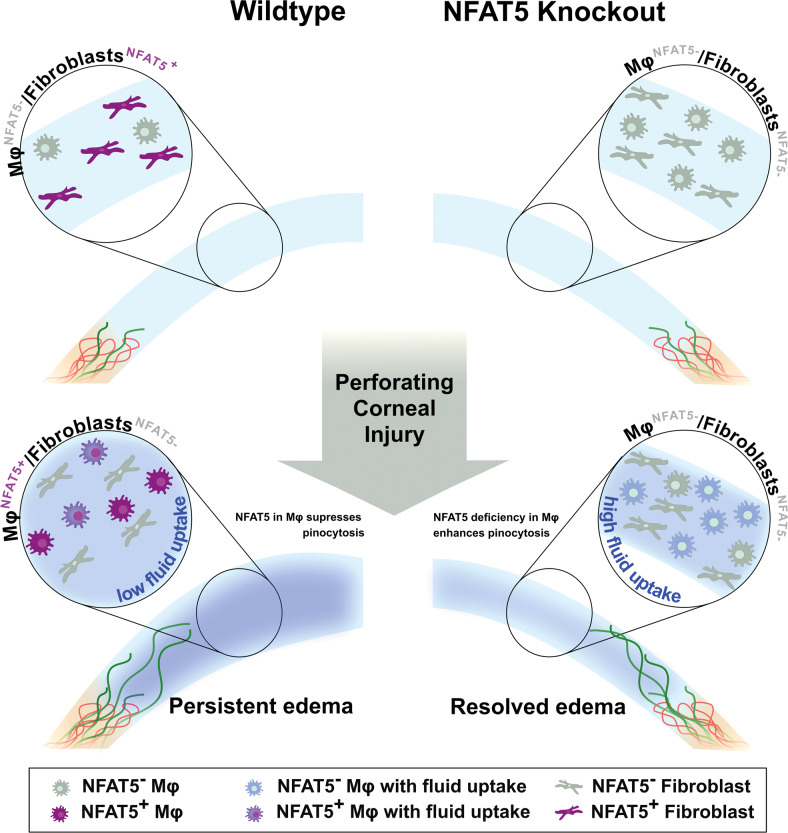
Presumed mechanism for the role of NFAT5 in acute corneal edema resorption after perforating corneal injury (PCI). The naive (wild-type) cornea expresses NFAT5 mainly in vimentin^+^ fibroblasts (purple), whereas the majority of corneal macrophages are NFAT5^-^ (gray). Blood (red) and lymphatic (green) vessels are present at the limbus. After PCI in wild-type mice, NFAT5 is mainly expressed by corneal macrophages (purple). In wild-type corneas, macrophage pinocytosis (blue) is low, resulting in persistent corneal edema. In contrast, macrophages in mice defective for NFAT5 show higher pinocytosis capacity, leading to a faster resorption of corneal edema after PCI; lymphatic vessels are unaltered.

Based on the findings of this study, we propose that loss of NFAT5 results in higher numbers of corneal macrophages with increased pinocytotic capacity, leading to a faster resolution of corneal edema after injury (Fig. 5).

The modulation of corneal NFAT5 might be of great clinical relevance, as corneal edema is one of the leading causes of corneal blindness worldwide. In addition to acute corneal edema, which was investigated in our study, chronic corneal edema, as in Fuchs endothelial corneal dystrophy, is one of the major indications for corneal transplantation worldwide (~50% in 2016)^33^. Furthermore, the modulation of NFAT5 might be a therapeutic avenue for patients suffering from hydrops in keratoconus to prevent massive corneal edema and to avoid surgical therapy^34^. Therefore, the availability of a nonsurgical approach to reduce corneal edema would be of great therapeutic value to minimize the shortage and costs of corneal transplants and to treat corneal blindness worldwide.

## Supplementary information

Supplemental Material

## References

[1] Cursiefen C (2006). Nonvascular VEGF receptor 3 expression by corneal epithelium maintains avascularity and vision. Proc. Natl Acad. Sci. USA.

[2] Congdon NG, Friedman DS, Lietman T (2003). Important causes of visual impairment in the world today. JAMA.

[3] Garg P, Krishna PV, Stratis AK, Gopinathan U (2005). The value of corneal transplantation in reducing blindness. Eye (Lond.).

[4] Cursiefen C (2007). Immune privilege and angiogenic privilege of the cornea. Chem. Immunol. Allergy.

[5] Folkman J, Shing Y (1992). Angiogenesis. J. Biol. Chem..

[6] Kato T (2001). Diminished corneal angiogenesis in gelatinase A-deficient mice. FEBS Lett..

[7] Beck L, D’Amore PA (1997). Vascular development: cellular and molecular regulation. FASEB J..

[8] Hos D (2016). IL-10 indirectly regulates corneal lymphangiogenesis and resolution of inflammation via macrophages. Am. J. Pathol..

[9] Hos D (2017). Transient ingrowth of lymphaticvessels into the physiologically avascular cornea regulates corneal edema and transparency. Sci. Rep..

[10] Kiesewetter A, Cursiefen C, Eming SA, Hos D (2019). Phase-specific functions of macrophages determine injury-mediated corneal hem- and lymphangiogenesis. Sci. Rep..

[11] Machnik A (2009). Macrophages regulate salt-dependent volume and blood pressure by a vascular endothelial growth factor-C-dependent buffering mechanism. Nat. Med..

[12] Wiig H (2013). Immune cells control skin lymphatic electrolyte homeostasis and blood pressure. J. Clin. Invest..

[13] Titze J (2004). Hypertension, sodium retention, calcium excretion and osteopenia in Dahl rats. J. Hypertens..

[14] Titze J (2003). Osmotically inactive skin Na+ storage in rats. Am. J. Physiol. Ren. Physiol..

[15] Titze J (2005). Internal sodium balance in DOCA-salt rats: a body composition study. Am. J. Physiol. Ren. Physiol..

[16] Machnik A (2010). Mononuclear phagocyte system depletion blocks interstitial tonicity-responsive enhancer binding protein/vascular endothelial growth factor C expression and induces salt-sensitive hypertension in rats. Hypertension.

[17] Choi SY, Lee-Kwon W, Kwon HM (2020). The evolving role of TonEBP as an immunometabolic stress protein. Nat. Rev. Nephrol..

[18] Kuper C, Beck FX, Neuhofer W (2014). Generation of a conditional knockout allele for the NFAT5 gene in mice. Front. Physiol..

[19] Clausen BE, Burkhardt C, Reith W, Renkawitz R, Forster I (1999). Conditional gene targeting in macrophages and granulocytes using LysMcre mice. Transgenic Res..

[20] Schey KL, Wang Z, J LW, Qi Y (2014). Aquaporins in the eye: expression, function, and roles in ocular disease. Biochim. Biophys. Acta.

[21] Snuggs JW (2021). TonEBP regulates the hyperosmotic expression of aquaporin 1 and 5 in the intervertebral disc. Sci. Rep..

[22] Lewis WH (1931). Pinocytosis. Bull. John Hopkins Hosp.

[23] Lewis WH (1937). Pinocytosis by malignant cells. Am. J. Cancer.

[24] Berry MR (2017). Renal Sodium gradient orchestrates a dynamic antibacterial defense zone. Cell.

[25] Cursiefen C (2011). Thrombospondin 1 inhibits inflammatory lymphangiogenesis by CD36 ligation on monocytes. J. Exp. Med..

[26] Su H (2021). Cancer cells escape autophagy inhibition via NRF2-induced macropinocytosis. Cancer Cell.

[27] Kappert L (2021). Loss of Nfat5 promotes lipid accumulation in vascular smooth muscle cells. FASEB J..

[28] Canton J (2016). Calcium-sensing receptors signal constitutive macropinocytosis and facilitate the uptake of NOD2 ligands in macrophages. Nat. Commun..

[29] Redka DS, Gutschow M, Grinstein S, Canton J (2018). Differential ability of proinflammatory and anti-inflammatory macrophages to perform macropinocytosis. Mol. Biol. Cell.

[30] Choi S (2017). Transcription factor NFAT5 promotes macrophage survival in rheumatoid arthritis. J. Clin. Invest..

[31] Neubert P (2019). HIF1A and NFAT5 coordinate Na(+)-boosted antibacterial defense via enhanced autophagy and autolysosomal targeting. Autophagy.

[32] Cipolla MJ, Crete R, Vitullo L, Rix RD (2004). Transcellular transport as a mechanism of blood-brain barrier disruption during stroke. Front. Biosci..

[33] Flockerzi E (2018). Trends in corneal transplantation from 2001 to 2016 in Germany: a report of the DOG-section cornea and its keratoplasty registry. Am. J. Ophthalmol..

[34] Bachmann B, Handel A, Siebelmann S, Matthaei M, Cursiefen C (2019). Mini-descemet membrane endothelial keratoplasty for the early treatment of acute corneal hydrops in keratoconus. Cornea.

