# Supplementary material for: The role of the osmosensitive transcription factor NFAT5 in corneal edema resorption after injury
Source: Exp Mol Med. 2023 Mar 3;55(3):565–73. doi: 10.1038/s12276-023-00954-w (PMC10073147; doi:10.1038/s12276-023-00954-w)
Supplement: Supplementary file 1 — Supplemental Material [file 12276_2023_954_MOESM1_ESM.pdf]

Supplementary Material

Genotyping Protocols

NFAT5 flox

PCR reaction mix

BioBudget Master Mix	4 µl
Each Primer	0.5 µl
H_2O	13.5 µl
DNA	1 µl

Cycler Program:

95 °C	15 min	
95 °C	45 sec	35 x
59 °C	60 sec	
72 °C	60 sec	
72 °C	7 min	
10 °C	∞	

Primer Sequences:

NFAT5-Lox2:

5′-GTA ACC ATG ATT AGT CTT TTA GCT TTA TG-3′

NFAT5-SDL2

5′-GTT CTG AGA ATC CAA AGC ACA AC-3′

Expected bands:

Wildtype: 334 bp
Flox: 392 bp

NLSCre (for LysMCre and UbcCre)

PCR reaction mix

BioBudget Master Miy	4 µl
Each Primer	0.5 µl
H_2O	14 µl
DNA	1 µl

Cycler Program:

95 °C	15 min	
95 °C	45 sec	30 x
59 °C	60 sec	
72 °C	60 sec	
72 °C	7 min	
10 °C	∞	

Primer Sequences:

NLSCre UP:

5´ GGA AAT GGT TTC CCG CAG AAC CTG A 3´

NLSCre DO:

5´ GAT GAG TTG CTT CAA AAA TCC CTT CCA 3´

Expected bands:

Wildtype: none
Cre: 630 bp

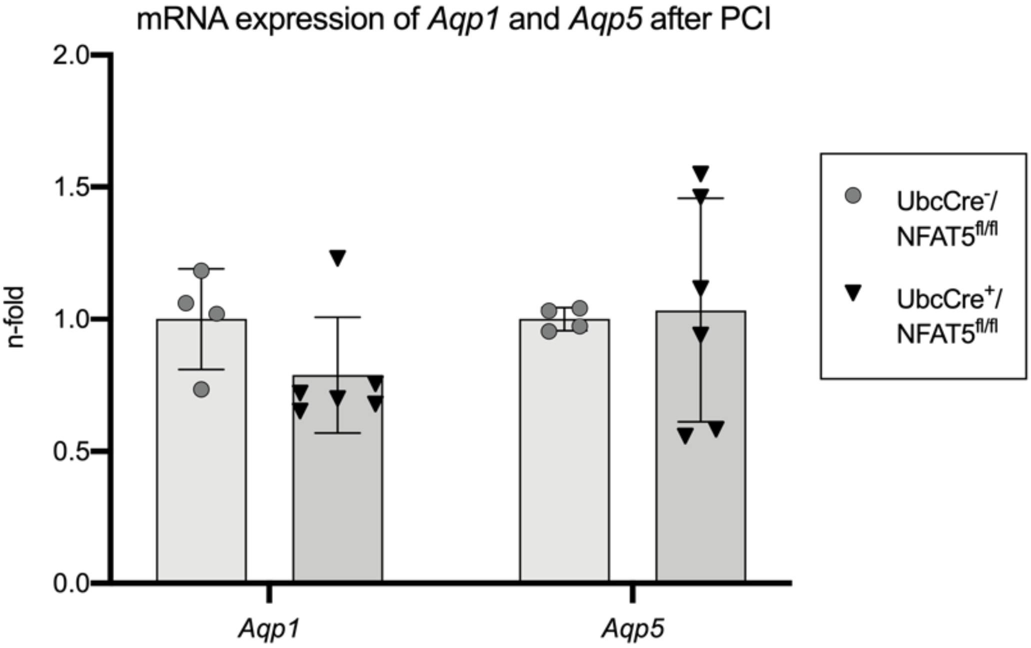

Supplementary Fig. 1 : mRNA expression of Aquaporin 1 and 5 is not altered in corneas after PCI in UbcCre^{+}/NFAT5$^{fl/fl}$ mice compared to UbcCre^{-}/NFAT5$^{fl/fl}$ mice.
